# Genome-Wide Identification and Functional Analysis of NAP1 in *Triticum aestivum*

**DOI:** 10.3390/genes14051041

**Published:** 2023-05-04

**Authors:** Huimin Feng, Mila Wu, Ziqiong Wang, Xia Wang, Jianping Chen, Jian Yang, Peng Liu

**Affiliations:** 1 State Key Laboratory for Managing Biotic and Chemical Threats to the Quality and Safety of Agro-Products, Institute of Plant Virology, Ningbo University, Ningbo 315211, China; 2 Key Laboratory of Biotechnology in Plant Protection of MARA and Zhejiang Province, Institute of Plant Virology, Ningbo University, Ningbo 315211, China

**Keywords:** NAP1, wheat, plant immunity, plant gene expression, plant viruses

## Abstract

As a main molecular chaperone of histone H2A-H2B, nucleosome assembly protein 1 (NAP1) has been widely researched in many species. However, there is little research investigating the function of NAP1 in *Triticum aestivum*. To understand the capabilities of the family of NAP1 genes in wheat and the relationship between TaNAP1 genes and plant viruses, we performed comprehensive genome-wide analysis and quantitative real-time polymerase chain reaction (qRT-PCR) for testing expression profiling under hormonal and viral stresses. Our results showed that TaNAP1 was expressed at different levels in different tissues, with higher expression in tissues with high meristematic capacity, such as roots. Furthermore, the TaNAP1 family may participate in plant defense mechanisms. This study provides a systematic analysis of the NAP1 gene family in wheat and lays the foundation for further studies on the function of TaNAP1 in the response of wheat plants to viral infection.

## 1. Introduction

Histone chaperones are proteins that combine DNA and histones to form nucleosome structures and disassemble intact nucleosomes into their subcomponents [1,2]. As negatively charged proteins, they prevent the misaggregation of negatively charged DNA and positively charged histones. Furthermore, histone chaperones play an essential regulatory role in the complex steps of histone and DNA folding to form properly assembled nucleosomes and are involved in the modulation of gene expression in the development process of many plants [1,2,3]. Nucleosome assembly protein 1 (NAP1) is a member of the histone chaperone family and an indispensable element of eukaryotic chromatin construction, maintenance, and animation [1]. NAP1 delivers histones, which assemble nucleosomes and facilitate the movement of nuclear proteins, into the nucleus, affecting many genes’ transcription [1,4,5,6]. NAP1 was originally purified and identified from the eggs of *Xenopus laevis*, was subsequently separated from mouse and human cells, and is evolutionarily conserved among different species [2]. In HeLa cells, NAP1 was shown to cooperate with the novel synthetic histones H2A and H2B in vitro; it was also identified in yeast as a subcomponent of the histone variant H2A or Z-specific exchange complex SWR1 [7]. In plants, NAP1 was first cloned in soybeans and was shown to possess the same nucleosome assembly function [8]. Subsequently, NAP1 genes were identified in other plants such as *Arabidopsis thaliana, Nicotiana tabacum,* and *Oryza sativa* [9,10]. Four homologous NAP1 proteins, NAP1:1–NAP1:4, and two distantly associated proteins called NAP1-related proteins (NRP1 and NRP2) belong to the same family in both *O. sativa* and *A. thaliana*. Similar to NAP1, NRPs also contain a conserved structural domain; however, they are phylogenetically and structurally distinct from NAP1 [10,11,12].

Previous studies in Arabidopsis showed that the absence of NRP or NAP1 subfamily proteins resulted in reduced homologous recombination in plants under normal growing circumstances, as well as under extensive genotoxic or abiotic stress. Concurrent knockout of *NRP1* and *NRP2* genes results in the suppression of postembryonic root growth and disturbs the expression of some genes. Plants mutated with NAP1 are hypersensitive to genotoxic stress, showing elevated levels of transcriptional gene silencing and DNA damage [11]. Notably, the mutant plants were more susceptible to necrosis caused by pathogens, while the overexpression lines were more tolerant to pathogen infection, which correlated with their adult and juvenile characteristics, respectively [13]. Collectively, the deletion of NAP and NRP subfamily members would result in abnormal plant growth and development as well as susceptibility to pathogens, further suggesting that the NAP1 family serves an important part in plant innate immunity and growth.

*T. aestivum* is one of the main food crops globally and is widely grown in Europe, Asia, North America, and other places where it is suitable for cultivation. Wheat production is a matter of food security worldwide. Its yield can be affected by various biological and non-biological factors, including the environment and climate change, insect herbivory, and fungal and viral diseases [14]. Viral diseases affecting wheat yield have been reported in many countries, especially in Europe, North America, and Asia. One of the major pathogens causing wheat mosaic disease was *Chinese wheat mosaic virus* (CWMV) [15]. CWMV was first found in winter wheat in Shandong at the end of the last century, can cause yield losses of 10–30% and up to 70% in severe cases [16]. CWMV is a member of the genus *Furovirus*, family *Virgaviridae*, which possess a rod-shaped viral particle and a genome comprising two single-stranded RNAs, named RNA1 and RNA2 [17]. CWMV RNA1 is 7147 nt in size and contains three open reading frames (ORF) encoding three proteins, of which ORF1 encodes a 153 kDa polypeptide with a UGA-stop codon that can be read through ORF2 to produce an RNA-dependent RNA polymerase (RdRp) of approximately 212 kDa. ORF3 encodes a 37 kDa movement protein [18]. CWMV RNA2 is 3569 nt in size and contains three ORFs that encode four proteins. ORF1 encodes a 19 kDa coat protein (CP) whose stop codon UGA can partially be read through the ORF to produce an 84 kDa CP-RT protein. A CUG codon preceding the 19 kDa CP start codon AUG can also be used as the start codon for transcription, producing a protein of approximately 23 kDa with 40 more amino acids at its N-terminal than the normal CP protein, called N-CP. ORF3 encodes a cysteine-rich protein (CRP) of approximately 19 kDa [16,19,20].

The NAP1 gene family is essential for the physiological activities of various plants and plays an important role in the immunity of plants. However, the structural features and phylogenetic relationships of the NAP1 family in wheat have not been characterized. In this study, 13 NAP1 homologs in wheat genome were identified and classified into two categories based on the characteristics of their structural domains. The replication relationships, chromosomal loci, protein motifs, exon–intron structures, protein structures, and subcellular locations of TaNAP1 were analyzed. The expression pattern of TaNAP1 homologs were analyzed under different tissue, hormone, and CWMV infection conditions. The aim of this study was to build the foundation for further functional studies of NAP1 in the wheat defense response against viral infections.

## 2. Materials and Methods

### 2.1. Identification of the Whole Genome of the TaNAP1 Family

To identify the NAP1 protein family in wheat, we used the NAP1 proteins of *A. thaliana* (AtNAP1) as BALSTP templates. The *Arabidopsis* NAP1 protein sequence files were downloaded from the Information Resource Center of Arabidopsis (https://www.arabidopsis.org/, accessed on 14 October 2022). Genomic data of wheat were acquired from the Ensembl Plants Database (https://plants.ensembl.org/Triticum_aestivum/Info/Index, accessed on 14 October 2022). NAP1 homologs in wheat were screened using a stringency of <1 × 10^−6^ and ID% > 50 as the cut off. By submitting the putative TaNAP1 protein sequence, the potential members of the TaNAP1 gene family were validated using Pfam (https://pfam.xfam.org/, accessed on 14 October 2022).

### 2.2. Physical and Chemical Properties of TaNAP1 Genes

Additional information about the TaNAP1 gene family, such as the counts of amino acids, chromosomal localization, and length of coding sequences, was obtained from the Ensembl Plant database. The molecular weight and theoretical isoelectric point of each TaNAP1 protein were obtained using ExPAsy (https://web.expasy.org/compute_pi/). Cell-PLoc 2.0 (http://www.csbio.sjtu.edu.cn/bioinf/Cell-PLoc-2/, accessed on 15 October 2022) which was used to predict subcellular localization.

### 2.3. Multiple Sequence Alignments and Phylogenetic Tree Construction

Phylogenetic analysis was performed using sequences from three datasets: the identified TaNAP1 protein sequence, 6 AtNAP1 protein sequences downloaded from The Arabidopsis Information Resource (https://www.arabidopsis.org/, accessed on 15 October 2022), and 11 NAP1 proteins from *O. sativa* (OsNAP1) that were downloaded from the Rice Genome Annotation Project (http://rice.plantbiology.msu.edu/downloads_gad.shtml, accessed on 15 October 2022). Multiple sequence alignment was conducted through a MUSCLE function using MEGA-11 (Version:11.0.13) software. Moreover, the phylogenetic tree based on 1000 bootstrap replications was generated using the neighbor-joining approach, and the p-distance method and pairwise deletion alternative were used to address gaps in the amino acid sequence [21,22]. Next, a systemic phylogenetic tree of TaNAP1 protein sequences was constructed using the same approach.

### 2.4. The Chromosomal Location, Synteny Analysis, and Duplication of TaNAP1

The wheat genome sequence and genome comment files were obtained from the Ensembl Plant database. The MCScanX function of TBtools software(Version:1.098768) was used to generate chromosomal localization of NAP1 in wheat, gene duplication events in the wheat genome, and synthetic relationships between wheat and other species [23]. Gene duplication events of NAP1 within the wheat genome and synthetic relationships between wheat and other species were analyzed using TBtools MCScanX.

### 2.5. Calculation of Ka/Ks Values

The Ka/Ks value is a powerful indicator of selection pressure. The Ka/Ks calculator function of TBtools software(Version:1.098768) was used to calculate Ka and Ks values [24]. In addition, we used the equation T = Ks/(2 × 9.1 × 10^−9^) million years ago (Mya) to calculate the divergence time (T) [25].

### 2.6. Structural Analysis of the TaNAP1 Gene and Predicted Tertiary Structure of Proteins

Predictions of TaNAP1 patterns were performed using the MEME Suite 5.2.3 online analysis site (http://alternate.meme-suite.org/tools/meme, accessed on 15 October 2022). Predictions resulted in 10 conserved motifs. The genome and coding sequence sequences of TaNAP1 were used to determine the structure of gene exons using online site (http://gsds.gao-lab.org/, accessed on 15 October 2022) [26]. All results were rearranged using TBtools [23]. The SWISS-MODEL library of templates is a large database of structures containing experimentally established protein structures [27]. Homology modeling was performed by SWISS-MODEL and we obtained the predicted 3D structure of TaNAP1 [28].

### 2.7. Tissues Expression Profiles of TaNAP1

Measuring the expression levels of five randomly selected TaNAP1 genes among five various tissues. Samples from three replicates of each wheat tissue were taken and stored at −80 °C until total RNA was derived. Individual gene expression levels were measured by quantitative real-time polymerase chain reaction (qRT-PCR). Heat maps and analysis results of tissue-specific expression profiles were created using TBtools [23].

### 2.8. Cis-Acting Element Prediction

All promoter sequences, including the 2000 bp upstream of the TaNAP1 gene, were downloaded from the Ensembl Plants Database [29]. Using the PlantCARE (http://bioinformatics.psb.ugent.be/webtools/plantcare/html/, accessed on 15 October 2022) database, calculations predicted the conserved cis-acting elements present in the identified region of promoter [30]. Heat maps of analysis results were exhibited using TBtools together with promoter element analysis for hormone reactions.

### 2.9. Expression of TaNAP1 under Abscisic Acid and Methyl Jasmonate Treatments

Abscisic acid (ABA) and methyl jasmonate (MeJA) were used to treat wheat plants at the three-leaf stage to analyze the changes in NAP1 expression levels. Treatment of wheat seedlings with 100 μmol L^−1^ of either ABA or MeJA [31]. Wheat treated with distilled water was used as a negative control. At four different time points (2, 4, 6, and 12 h) samples were collected and collected at −80 °C until the RNA of the total sample needs to be extracted. Detection of gene expression levels was performed using qRT-PCR.

### 2.10. Expression of TaNAP1 after CWMV Infection

Two groups of wheat cultured under the same circumstances were selected and three biological replicates of each group were chosen for gene expression analysis. Transcription and frictional inoculation of CWMV were performed as previously described [32]. Untreated wheat samples were used as negative controls. Seven days after inoculation with CWMV, wheat leaves were gathered for qRT-PCR analysis. Four TaNAP1 genes were randomly selected for investigation of the variations in gene expression levels after viral infection.

### 2.11. Expression Analysis of TaNAP1 by qRT-PCR

Using HiPure Plant RNA Mini Kit (Magen, Guangdong, China), total RNA was extracted from each sample according to the instructions and store at −80 °C. Synthesize first-strand cDNA from total RNA using the First Strand cDNA Synthesis Kit ReverTra Ace-α-™ (TOYOBO, Osaka, Japan) [33]. Gene expression levels were analyzed using an RT-PCR machine (ABI) with a qPCR SYBR green master mix. The program used for qRT-PCR was 5 min at 95 °C, followed by 40 cycles of 15 s at 95 °C, 20 s at 62 °C, and 30 s at 72 °C. All primers for qRT-PCR were designed using Primer-BLAST of NCBI (http://www.ncbi.nlm.nih.gov/tools/primer-blast, accessed on 15 October 2022). The *T. aestivum* cell division control protein (TaCDC) gene (accession number: TraesCS4D02G267600) [34] was used as an internal reference gene. The relative expression levels of TaNAP1 were calculated using the ΔΔCt method [35].

### 2.12. Plant Growth and TaNAP1 Subcellular Localization Assay

The germination of wheat cv. YangMai 158 and *Nicotiana benthamiana* plants was performed in an artificial culture room at 25 °C, relative humidity of 65 ± 5%, and long-daylight conditions (16 h light/8 h dark cycle) [36]. Plants were placed in growth cabinets at different temperatures (8, 15, 20, and 25 °C) with a 16 h light/8 h dark photoperiod for the temperature stress treatment. Plants placed at 8 °C served as controls. We cloned the coding sequences of TaNAP1:6 and TaNAP1:11 constructed a transient expression vector with a green fluorescent label. The construct was briefly transformed into *Agrobacterium tumefaciens* (strain GV3101) by electroporation. The transformants were incubated in inoculation buffer (10 mM MgCl_2_, 100 mM MES, 2 mM acetosyringone [pH 5.7]) and resuspended at room temperature for 3–5 h. The suspension was then adjusted to OD600 = 0.1 and infiltrated into the leaves of 4–6 weeks-old H2B-RFP transgenic *N. benthamiana* plants using a needleless syringe. After 60 h, infiltrated leaves were collected and observed under a confocal microscope (LEICA).

## 3. Results

### 3.1. Identification and Characterization of NAP1 in Triticum aestivum

In previous studies, there are six NAP1 proteins in Arabidopsis and seven in rice. A BLASTP search was performed on wheat genomic data using the NAP1 amino acid sequences of Arabidopsis and rice as a query. As a result, 13 NAP1 members were identified and named TaNAP1:1 through TaNAP1:13. The details of the TaNAP1 gene family, including gene ID, chromosome location, groups, and physicochemical properties, are provided in Table 1.

### 3.2. Phylogenetic Analysis of TaNAP1 and the Tertiary Structure Models

**Figure 1:**
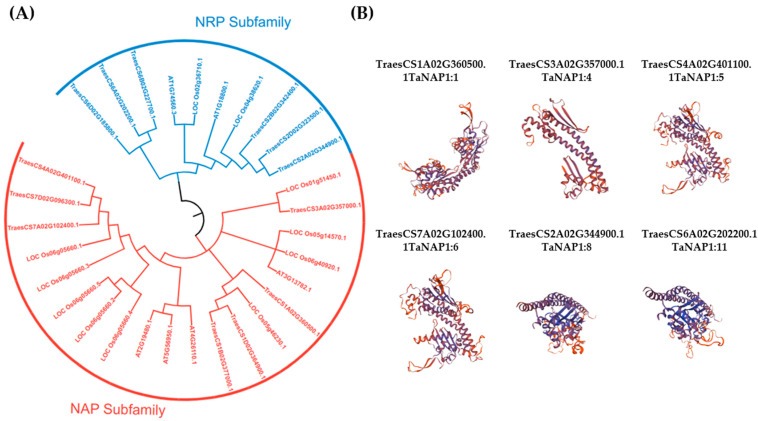
Phylogenetic analysis of TaNAP1 and the tertiary structure models. (**A**) A phylogenetic tree was constructed in MEGAX based on the full-length amino acid sequences of NAP1 proteins from *A. thaliana*, *O. sativa*, and *T. aestivum* with 1000 bootstrap replicates using ClustalW. The tree is divided into two subfamilies, the NRP subfamily is blue and the NAP subfamily is red. (**B**) Structure prediction using SWISS-MODEL; four of the selected proteins belonged to the NAP subfamily and two belonged to the NRP subfamily, based on QMEAN and GMQE, the model with the optimum results was chosen.

Aiming to analyze the phylogenetic relationships between NAP1 from various species, the NAP1 protein sequences from *A. thaliana*, *O. Sativa*, and *T. aestivum* were used to construct a neighbor-joining phylogenetic tree using MEGA-X. Based on the classification and conserved structure of NAP1 in *O. sativa* and *A. thaliana* [21], TaNAP1 can be classified into two subfamilies: NRP and NAP (Figure 1A). All three species contain members of both subfamilies, demonstrating that the subfamilies are present in both dicotyledons and monocotyledons. The NAP subfamily has seven members, including TaNAP1:1, TaNAP1:2, TaNAP1:3, TaNAP1:4, TaNAP1:5, TaNAP1:6, and TaNAP1:7, whereas the NRP subfamily has six members, including TaNAP1:8, TaNAP1:9, TaNAP1:10, TaNAP1:11, TaNAP1:12, and TaNAP1:13 (Table 1).

To gain insight into the structural effects of the TaNAP1 protein, we generated a 3D protein model of TaNAP1 using the homology model available in the SWISS-MODEL template library. Four NAP and two NRP subfamilies were randomly selected and displayed. The results showed that proteins within the same subfamily had similar tertiary structures, while tertiary structures differed significantly between the two subfamilies. This revealed the structural diversity of the TaNAP1 family in terms of protein structure (Figure 1B).

### 3.3. Genetic Structure and Analysis of Conserved Patterns of NAP1

**Figure 2:**
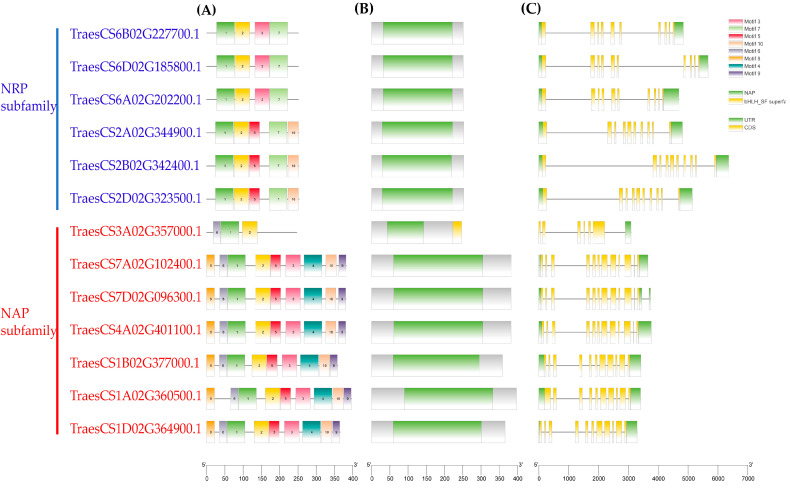
Analysis of the gene structure of TaNAP1. (**A**) Conserved mods of the TaNAP1 protein family, with motif1-motif 10 displayed in different colored boxes. (**B**) Conserved domain of the TaNAP1 protein family. (**C**) Gene structure of the *TaNAP1* gene family, CDS (yellow rectangle), UTR (green rectangle), and introns (grey line). The sequence length is displayed at the bottom of the graph.

Identical genes with different structures play important roles in the evolution of species. Thus, we analyzed the gene structure, conserved domains, and conserved motifs of TaNAP1 (Figure 2). Our results showed that *TaNAP1* members shared a similar genetic structure with some differences. This raises the possibility of a certain degree of functional diversity among the genes in this family. The number of exons in the *TaNAP1* genes mostly ranged from ten to twelve, with a maximum of twelve (*TaNAP1:5*, *TaNAP1:6*) and a minimum of six (*TaNAP1:4*) (Figure 2C). The MEME online analysis tool was used to identify common patterns between the NAP1 proteins within the two groups and the findings were presented using TBtools (Figure 2B). Consequently, ten conservative patterns were identified. The patterns are distributed in the NAP subfamily as shown in the figure (Figure 2A). Identical groups of NAP1 proteins displayed a similar distribution of conserved patterns. All members of the NRP subfamily contained motifs 1, 2, and 7, while some contain motifs 3, 5, and 10. Members of the NAP subfamily contain motifs 1, 2, and 6. All other members of the NAP subfamily contained all motifs except motif 7. Some motifs exist only in specific subfamilies, such as motif 7 in the NRP subfamily and motifs 4, 6, 8, and 9 in the NAP subfamily.

### 3.4. The Chromosomal Location, Synteny Analysis, and Duplication Events of TaNAP1

**Figure A1:**
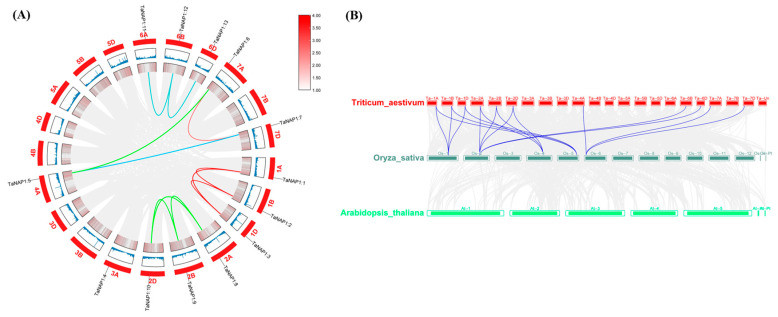
Synteny analysis and chromosome location of NAP1 genes in *T. aestivum*. (**A**) Synteny Analysis of TaNAP1, Colored lines indicate duplicated NAP1 gene pairs, gray lines represent the synthesis result of the *T. aestivum* genome, and the blue line in the box represents the density of genes on the chromosome. The TaNAP1 family chromosomes localized in the outermost circle show. (**B**) Analysis of NAP1 gene synthesis in *T. aestivum*, *O. sativa* and *A. thaliana*. The gray line in the background represents the syntonic blocks of *T. aestivum* with *O. sativa* and *A. thaliana*, and the red line represents the syntonic NAP1 gene pairs.

Since wheat is hexaploid and contains three subgenomes (A, B, and D), three homologs from homologous chromosomes are possible for each wheat gene. The results showed that all *TaNAP1* members were evenly distributed on 13 chromosomes, with three *TaNAP1* genes on chromosomes 1, 2, and 6, two on chromosome 7, and only one on chromosomes 3 and 4. Three *TaNAP1* members on chromosomes 1, 2, and 6 were evenly distributed on subgenomes A, B, and D, whereas the two *TaNAP1* members on chromosome 7 were distributed on chromosomes 7A and 7D. *TaNAP1* members on chromosomes 3 and 4 were distributed on chromosomes 3A and 4A (Figure A1). To understand the situation in wheat, we assessed in Circos the tandem repeat events of the *TaNAP1* family to establish the chromosomal location and replication relationships of all *TaNAP1* genes (Figure A1A). A total of 11 segmental duplicate pairs were identified among the 13 *TaNAP1* members in *T. aestivum*. The homologous duplicate gene pairs we identified were all formed by segmental duplication or whole-genome duplication. Furthermore, we calculated the substitution rates of nonsynonymous substitutions (Ka) and synonymous substitutions (Ks) for each homologous pair to investigate the duplication patterns of the *TaNAP1* genes during evolution. The Ka/Ks ratio ranged from 0 to 0.3231 and all duplicated *TaNAP1* gene pairs had Ka/Ks ratios of less than 1. Based on the divergence rate of 9.1 × 10^−9^ synonymous mutations per synonymous locus per year, the equation T = Ks/(2 × 9.1 × 10^−9^) Mya was used to evaluate the divergence time (T). The 11 duplicated *TaNAP1* member pairs diverged 0.725–7.242 Mya (Table A1). We also performed the synthetic analysis of the evolutionary relationship of *TaNAP1* members among *T. aestivum*, *O. sativa*, and *A. thaliana*. The results showed that 17 homologous gene pairs were found between *O. sativa* and *T. aestivum* (Figure A1B). The *TaNAP1* members in *T. aestivum* were mainly orthologous to Chr1, Chr2, Chr4, Chr5, and Chr6 in *O. sativa*. *TaNAP1* members located on Chr1 and Chr2 (including three subgenomes) have two orthologous pairs in *O. sativa*, whereas TaNAP1 genes located on Chr4A, Chr6B, Chr6D, Chr7A, and Chr7D have only one orthologous pair in *O. sativa*. The analysis showed that the TaNAP1 members located on Chr3A and Chr6A had no orthologous pair in *O. sativa*. A notable orthologous relationship in the *TaNAP1* members could be found between *T. aestivum* and *O. sativa*, whereas no orthologous relationship was found in *A. thaliana*.

### 3.5. Analysis of the TaNAP1 Promoter Region

**Figure 3:**
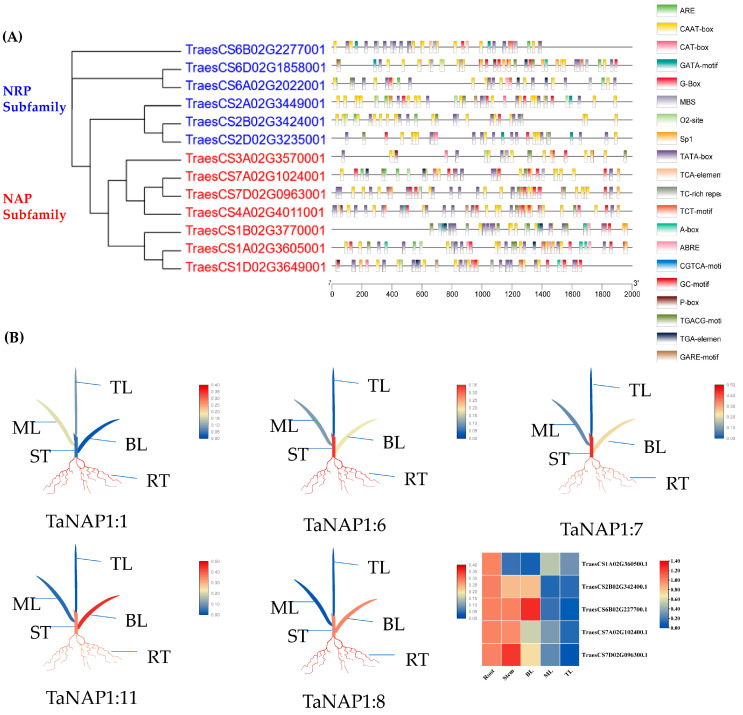
Prediction of *TaNAP1* gene cis-acting elements and differential tissue-specific expression of TaNAP1. (**A**) *TaNAP1* genes are shown on the left; different colors belong to different subfamilies; the tick at the foot indicates the promoter sequence length; different colored boxes indicate different cis-acting elements; the name of the cis-acting element is shown on the right. (**B**) Three independent biological replicates were used to calculate the average expression levels of *TaNAP1* in other tissues relative to the root and visualize the results in TBtools. Red squares represent high expression levels; blue squares represent low expression levels.

The promoter regions (2000 bp upstream of the translation start site) of 13 *TaNAP1* members were analyzed using the PlantCARE database to decipher the role of the cis-acting elements of the TaNAP1 gene in response to biotic and abiotic stresses (Figure 3A). After analyzing the results, we identified 20 cis-acting elements, which predominantly associated with abiotic stress response, growth and development, hormone response, and light response.

### 3.6. Expression Profile of TaNAP1 in Wheat at the Three-Leaf Stage

For a comprehensive understanding of *TaNAP1* function, the expression of five *TaNAP1* members randomly selected from two subfamilies (two belonging to NRP and three from NAP) was analyzed by qRT-PCR in five various tissues (top leaves, middle leaves, bottom leaves, roots, and stems). The results showed that the five *TaNAP1* members were expressed in all the wheat tissues (Figure 3B). All genes showed high expression levels in the roots. Except for *TaNAP1:1*, the expression levels of *TaNAP1* members in the plant stems were analogous to that in the roots, while the expression level was low in the leaves. *TaNAP1:1* was expressed at low levels in all tissues except the roots.

### 3.7. Analysis of TaNAP1 Expression under Different Stresses

**Figure 4:**
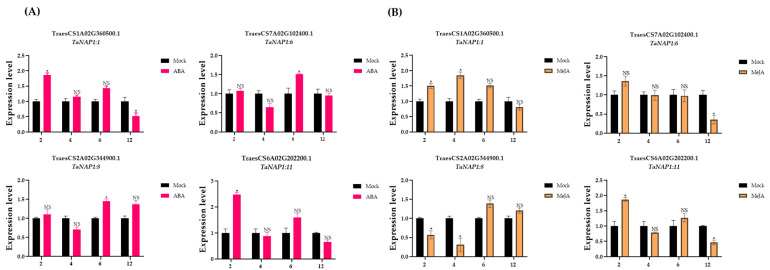
The expression level of the *TaNAP1* gene under hormone stress. Wheat leaves were sprayed with two hormones, ABA (**A**) and MeJA (**B**). Each treatment was subjected to three biological replicate experiments and and detected gene expression by qRT-PCR. The relative expression levels of the four TaNAP1 genes in response to the plant hormones (ABA and MeJA) on wheat seedling leaves at 2, 4, 6, and 12 h. Analysis of gene expression data was carried out using Excel(Version:16.0.16227.20280) software and visualized with GraphPad Prism(Version:9.0.0) software. Differences with statistical significance (*p* < 0.05; one-way ANOVA) are represented by an asterisk (*) and bars mean standard deviations (±SD) calculated for three biological replicates. NS, no significance.

Since all *TaNAP1* members contain hormone regulators, we explored the role of the relative expression level of TaNAP1 members in hormone responses. Four *TaNAP1* genes with hormone regulatory sites (ABA and MeJA) were selected and the influence of hormonal pressures on *TaNAP1* was verified. The results revealed that *TaNAP1:11* was very sensitive to both ABA and MeJA, whereas *TaNAP1:8* was less sensitive to ABA (Figure 4A) and more sensitive to MeJA (Figure 4B).

**Figure 5:**
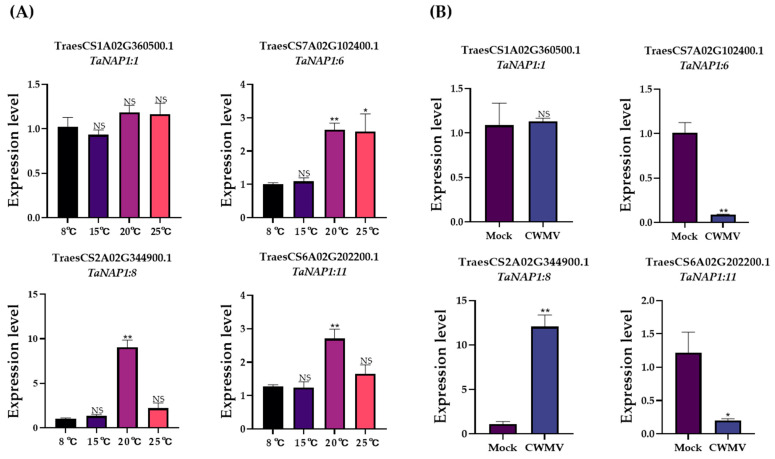
*TaNAP1* gene expression levels under different stresses. (**A**) Measuring the relative expression levels of *TaNAP1* in plants that were grown at various temperatures for 14 days by qRT-PCR. The average expression values from three independent biological replicates and three technical replicates were calculated. Data originating from the 8 °C treatment group were used as controls. (** *p* < 0.01; * *p* < 0.05; NS, *p* > 0.05). (**B**) The expression levels of TaNAP1 gene after CWMV infection. Changes in gene expression of wheat seedlings infected with CWMV for seven days and detected by RT-qPCR. Healthy wheat samples from the same batch were used as controls (** *p* < 0.01; * *p* < 0.05; NS, *p* > 0.05).

The development of wheat at different temperatures is associated with different relative expression levels of *TaNAP1*. *TaNAP1:6*, *TaNAP1:7*, and *TaNAP1:11* showed low expression levels at lower temperatures (8 °C and 15 °C) and high expression levels at higher temperatures (20 °C and 25 °C) (Figure 5A). *TaNAP1:1* maintained a relatively high expression level at all temperatures. Moreover, *TaNAP1* expression levels exhibited different changes under CWMV infection conditions. The relative expression levels of *TaNAP1:6* and *TaNAP1:11* were significantly reduced by CWMV infection, whereas *TaNAP1:8* expression levels significantly increased after viral infection. Notably, as with different temperature conditions, the transcript accumulation of *TaNAP1:1* was not significantly changed after viral infection (Figure 5B).

### 3.8. Subcellular Localization Analysis of TaNAP1

**Figure A2:**
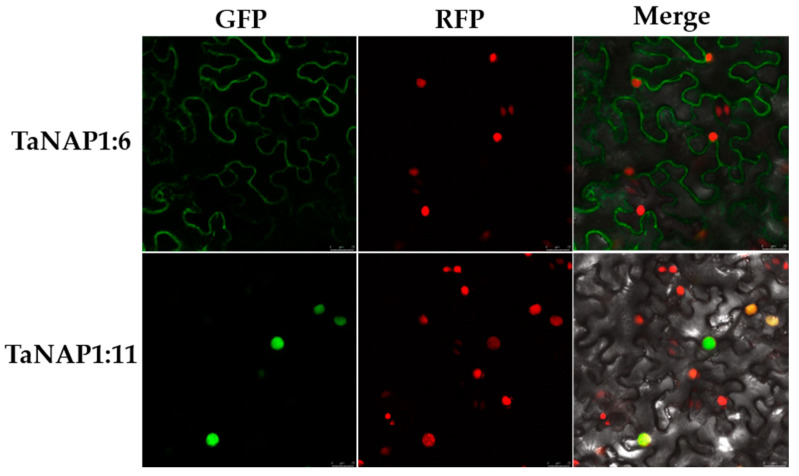
The subcellular localization of TaNAP1-GFP in epidermal cells of H2B-RFP transgenic *N. benthamiana* plants. Green fluorescence indicates the localization of TaNAP1 protein, and red fluorescence represents the nucleus. Confocal images were taken at 60 h post-infiltration (hpi). Scale bar = 25 μm.

Amino acid sequence analysis of TaNAP1 revealed that all TaNAP1 proteins were localized in the nucleus. To verify these predictions, we selected one TaNAP1 protein from each subfamily and analyzed its localization in H2B-RFP transgenic *N. benthamiana* plants using laser confocal microscopy. The results showed that TaNAP1:11 was localized in the nucleus, which was consistent with the predicted result, whereas TaNAP1:6 was localized in the cytoplasm, which was inconsistent with the predicted result (Figure A2).

## 4. Discussion

Histones and histone chaperones play significant roles in the modulation of the cell cycle, genomic stability, gene expression, and the response to biotic and abiotic stresses [37]. The NAP1 gene family is a crucial member of the histone chaperones. As H2A/H2B chaperones, they play a key role in the regulation of nucleosome structure and are involved in the regulation of chromatin deposition and flowering. The NAP1 family has been identified in several species including *X. laevis*, yeast, *Mus musculus*, *A. thaliana*, *N. tabacum*, *Phyllostachys edulis*, and *O. sativa* [2,9,10,38]. Nevertheless, to date, no member of the NAP1 gene family has been systematically studied in wheat. We therefore performed a basic genome-wide analysis of the NAP1 family in wheat and tentatively investigated the effect of TaNAP1 in wheat resistance to viral infection. In this study, 13 NAP1 genes were identified in wheat and, by constructing an interspecies evolutionary tree and referring to the classification of the NAP1 family in *A. thaliana* [21], we divided the NAP1 family into two subfamilies: the NRP and NAP. When comparing the formation of the two subfamilies, the number of NRP subfamily members was six for wheat, two for *A. thaliana*, *O. sativa*, and *Zea mays*, and four for *P. edulis*; this is probably owing to the fact that wheat is hexaploid, whereas *A. thaliana*, *O. sativa*, and *Z. mays* are diploid and *P. edulis* is tetraploid [38]. Gene structure analysis results indicated that members belonging to the same subfamily were similar, but also showed some differences. This suggests that genes in the same subfamily may have the same function, but functional diversity is not excluded.

Gene duplication events have played a crucial role in the evolution of gene families. They can increase functional variety and improve gene structure by introducing new family members, while helping organisms adapt to various environments. Investigation of gene duplication events helps us to understand the evolution of wheat. There are three types of gene duplication events: whole-genome duplication, segmental duplication, and tandem duplication [39]. In our study, the main replication event that occurred in *TaNAP1* was genome-wide replication, followed by segmental replication, without tandem replication events. To verify whether there was evolutionary pressure on *TaNAP1* and to explore its evolutionary pattern, values of Ka, Ks, and Ka/Ks were calculated from each paralogous gene (Ta-Ta) [25]. The value of Ks indicated that the replication event occurred 0.725–7.242 Mya in wheat. Furthermore, the results showed that the Ka/Ks values of each *TaNAP1* paralogous duplicated gene pair were much less than 1, indicating that the *TaNAP1* gene pair have experienced strong selection for purification during development and would remain functionally invariant as a result of negative selection. Analysis of *TaNAP1* synthesis showed that wheat is related to monocotyledonous *O. sativa*, but not to dicotyledonous *A. thaliana*, a result that is aligned with the evolutionary association between dicotyledons and monocotyledons.

Furthermore, we predicted the cis-acting elements to elucidate the potential biological capabilities of *TaNAP1*. These results indicated that the types of cis-acting regulatory elements [40] were different for each *TaNAP1*. Among these, GATA-motif, TCT-motif, Sp1, and G-Box are light-responsive elements [41], which indicate that TaNAP1 may be involved in the photomorphogenesis of wheat. ARE and GC-motif are involved in anaerobic induction and anoxia-specific induction, respectively [42]. MBS is an MYB-binding site involved in drought induction [43]. The O_2_-site is a cis-acting element involved in the regulation of zein metabolism, and the CAT-box is a cis-acting element related to meristem expression. CAAT-box is a common cis-acting element in the promoter and enhancer regions. The TATA -box is a core promoter element around −30 of the transcription start site. A-box is a cis-acting regulatory element. TC-rich repeats are a cis-acting element participating in stress and defense responses [44]. The GARE-motif, CGTCA-motif, TGACG-motif, TGA-element, TCA-element, P-box, and ABRE cis-acting elements are all related to plant hormone responsiveness [45]. The TGA-element is an auxin-responsive element. The GARE-motif and P-box are cis-acting elements associated with the gibberellin response, while TGACG-motif and CGTCA-motif are cis-acting regulatory elements involved in the MeJA responsiveness, and ABRE and TCA-element are involved in the regulation of abscisic acid and salicylic acid, respectively. Almost all TaNAP1 proteins contain cis-acting regulatory elements associated with hormone regulation, which indicates that *TaNAP1* may be engaged in specialized regulatory mechanisms connected with a variety of stress responses such as abiotic and biotic stress responses.

Plant hormones play a vital role in plant responses to biotic stresses. Precisely, the plant response to biotic stress is controlled by hormonally regulated defense pathways such as salicylic acid (SA), ethylene (Et), jasmonic acid (JA) and ABA [46]. Previous studies have found that ABA mediates plant defense by increasing callus deposition, thus limiting virus movement. Transgenic tomato plants carrying *Tm-1* were resistant to *Tobacco mosaic virus* (TMV) and exhibited increased ABA levels compared to susceptible plants [47]. Moreover, significant inhibition of TMV infection was observed after exogenous application of asparagine to activate the salicylic acid signaling pathway in *N. benthamiana* [48]. Likewise, it has been shown that rhizobia-mediated JA induction can reduce the symptoms of *Cucumber mosaic virus* (CMV) infection in *Col-0* [49]. Overexpression of the transcription factor OsNF-YA in rice inhibits the JA signaling pathways, thereby suppressing the plant’s antiviral defense, while silencing the gene enhances the resistance of rice to the virus [50]. In our study, the transcript level of all *TaNAP1 genes* was significantly increased by ABA or MeJA treatment, suggesting that NAP1 is involved in wheat immunity via ABA and MeJA signaling pathways.

The genomes of plant viruses are relatively small and encode a limited number of proteins, thus most viral proteins have complex functions [51]. The virus needs to use host proteins to promote its own infection. For example, after infecting rice, rice streak virus (RSV) and southern rice black-streaked dwarf virus (SRBSDV) induce the expression of *OsNF-YA* family genes, thus suppressing host immune activation and promoting their own infection [50]. The NIb protein of wheat yellow mosaic virus (WYMV) can bind to m^6^A methyltransferase B (MTB) protein, increase the m^6^A level of RNA1 of WYMV, and stabilize viral RNA, thus promoting viral infection [52]. CWMV CRP was first reported as an RNA silencing suppressor [19]. Subsequent studies showed that during CWMV infection, phosphorylated CRP was able to interact with TaUBA2C and dissociate it from chromatin, disrupting its RNA and DNA binding activity, thereby inhibiting the TaUBA2C-mediated disease resistance responses and promoting virus infestation [36]. In our study, CRP was able to interact with TaNAP1; therefore, we hypothesize that TaNAP1 may be involved in the process of resistance to CWMV infection in wheat.

In this study, we did not conduct a particularly in-depth study on the relationship between NAP1 and CWMV infection in wheat; therefore, there is no clear conclusion about the role of NAP1 in the CWMV infection process. As a histone chaperone, we can consider histone H2A/H2B together with NAP1 and CWMV in the future, which can give us a more comprehensive view to study the effect of histones and histone chaperones on viral infection.

## 5. Conclusions

To our knowledge, this is the first study to identify and characterize NAP1, a major chaperone of histone H2A-H2B in wheat. Thirteen TaNAP1 members classified as two subfamilies (NRP and NAP) were identified. Based on the gene structure and conserved structural domains, the TaNAP1 family has been highly conserved during evolution. In this study, we performed a holistic genome-wide analysis of the conserved structural domains, gene structure and protein patterns of TaNAP1, its chromosomal location, replication relationships, and expression profiles, as well as responses to hormone and plant virus stress. Results from qRT-PCR demonstrated that TaNAP1 expression was tissue-specific, with high expression in tissues with high meristematic capacity, such as the roots. Our results may provide a basis for subsequent experiments.

## Figures and Tables

**Table 1:** Characterization of 13 predicted NAP1 proteins in *T. aestivum*.

Name	Gene ID	Exons	Gene Location	CDS Length (bp)	Size (aa)	MW (kDa)	PI	Protein Location
**TaNAP1:1**	TraesCS1A02G360500.1	10	1A-541632666-541636078	1194	397	44.6	4.35	Nucleus
**TaNAP1:2**	TraesCS1B02G377000.1	11	1B-608533160-608536582	1080	359	41.1	4.33	Nucleus
**TaNAP1:3**	TraesCS1D02G364900.1	11	1D-445542135-445545423	1101	366	41.7	4.36	Nucleus
**TaNAP1:4**	TraesCS3A02G357000.1	6	3A-604998850-605001934	744	247	28.3	7.05	Nucleus
**TaNAP1:5**	TraesCS4A02G401100.1	12	4A-675131549-675135314	1149	382	42.8	4.23	Nucleus
**TaNAP1:6**	TraesCS7A02G102400.1	12	7A-62879024-62882679	1152	383	43.1	4.25	Nucleus
**TaNAP1:7**	TraesCS7D02G096300.1	11	7D-58500798-58504536	1149	382	42.9	4.24	Nucleus
**TaNAP1:8**	TraesCS2A02G344900.1	10	2A-582637903-582642714	762	253	29.4	4.13	Nucleus
**TaNAP1:9**	TraesCS2B02G342400.1	10	2B-488198352-488204713	762	253	29.47	4.17	Nucleus
**TaNAP1:10**	TraesCS2D02G323500.1	10	2D-416401053-416406189	762	253	29.46	4.13	Nucleus
**TaNAP1:11**	TraesCS6A02G202200.1	10	6A-337723056-337727746	756	251	28.8	4.26	Nucleus
**TaNAP1:12**	TraesCS6B02G227700.1	10	6B-353554146-353558991	756	251	28.8	4.26	Nucleus
**TaNAP1:13**	TraesCS6D02G185800.1	10	6D-242216366-242222032	756	251	28.8	4.26	Nucleus

Bp: base pair, CDS: coding sequence, aa: amino acids, Da: Dalton MW: molecular weight, PI: isoelectric point.

## Data Availability

Not applicable.

## Appendix A

**Table A1:** Ks, Ka, and Ka/Ks values calculated for paralogous NAP1 gene-pairs (*T. aestivum—T. aestivum*).

Paralogous Pairs		Ka	Ks	Ka/Ks	T (Mya)
TraesCS1A02G360500.1	TraesCS1B02G377000.1	0.010514	0.110014	0.095572	6.044715
TraesCS1A02G360500.1	TraesCS1D02G364900.1	0	0.076105	0	4.181585
TraesCS1B02G377000.1	TraesCS1D02G364900.1	0.010515	0.131817	0.079771	7.242715
TraesCS2A02G344900.1	TraesCS2B02G342400.1	0.013223	0.0274	0.482603	1.505511
TraesCS2A02G344900.1	TraesCS2D02G323500.1	0.006588	0.020386	0.323159	1.120127
TraesCS2B02G342400.1	TraesCS2D02G323500.1	0.00659	0.034253	0.19239	1.882007
TraesCS4A02G401100.1	TraesCS7A02G102400.1	0.020113	0.08873	0.226677	4.875255
TraesCS4A02G401100.1	TraesCS7D02G096300.1	0.006641	0.061125	0.108645	3.358501
TraesCS6A02G202200.1	TraesCS6B02G227700.1	0.001668	0.013202	0.126352	0.725366
TraesCS6B02G227700.1	TraesCS6D02G185800.1	0.001668	0.02664	0.062615	1.463729
TraesCS7A02G102400.1	TraesCS7D02G096300.1	0.014472	0.084035	0.172211	4.617296

Ka: nonsynonymous substitutions, Ks: synonymous substitutions, T: divergence time.
